# (*S*)-2-[(2-Hy­droxy­benz­yl)aza­nium­yl]-4-(methyl­sulfan­yl)butano­ate

**DOI:** 10.1107/S1600536811016564

**Published:** 2011-05-11

**Authors:** Giovanna Brancatelli, Giuseppe Bruno, Francesco Nicoló, Loredana Canfora, Giuseppe Ruisi

**Affiliations:** a Dip. di Chimica Inorganica Chimica Analitica e Chimica Fisica, Universitá degli Studi di Messina, Via Salita Sperone 31, I-98166 Vill. S. Agata–Messina, Italy; b Dip. di Chimica Inorganica e Analitica Stanislao Cannizzaro, Università di Palermo, Viale delle Scienze, I-90128 Palermo, Italy

## Abstract

The zwitterionic title compound, C_12_H_17_NO_3_S, is a reduced Schiff base derived from (*S*)-*N*-(2-hy­droxy­benzyl­idene)methio­nine. An intra­molecular inter­action between the N—H and carboxyl­ate groups forms a roughly planar (r.m.s. deviation = 0.1405 Å) five-membered ring containing the H(N), N, Cα, C(carboxyl­ate) and O atoms in a penta­gonal conformation. In the crystal, a supra­molecular triangle-shaped motif is generated by mol­ecules held together by O—H⋯O and N—H⋯O hydrogen bonds.

## Related literature

For transition metal complexes containing *N*-(2-hy­droxy­benz­yl)-α-amino acids as ligands, see: Bandyopadhyay *et al.* (2006 ▶); Beltrán *et al.* (2002 ▶); Ganguly *et al.* (2008 ▶); Koh *et al.* (1996 ▶); Martell (1989 ▶); Maurya (2003 ▶); Nefkens & Zwanenburg (1985 ▶); Ritsma (1975 ▶); Shongwe *et al.* (1999 ▶); Sreenivasulu & Vittal (2004 ▶); Wilson (1990 ▶).
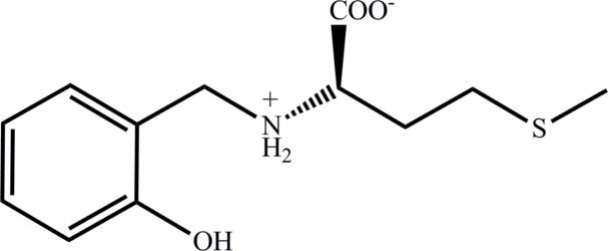


## Experimental

### 

#### Crystal data

C_12_H_17_NO_3_S
*M* *_r_* = 255.33
Triclinic, 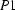

*a* = 5.3221 (2) Å
*b* = 5.8369 (2) Å
*c* = 10.5564 (5) Å
α = 98.200 (3)°
β = 90.780 (3)°
γ = 96.849 (3)°
*V* = 322.10 (2) Å^3^
*Z* = 1
Mo *K*α radiation
μ = 0.25 mm^−1^
*T* = 296 K
0.53 × 0.44 × 0.16 mm

#### Data collection

Bruker APEXII CCD diffractometer
Absorption correction: multi-scan (*SADABS*; Sheldrick, 1996 ▶) *T*_min_ = 0.679, *T*_max_ = 0.746
7430 measured reflections
2324 independent reflections
1966 reflections with *I* > 2σ(*I*)
*R*_int_ = 0.016

#### Refinement

*R*[*F* ^2^ > 2σ(*F* ^2^)] = 0.059
*wR*(*F* ^2^) = 0.179
*S* = 1.07
2324 reflections
156 parameters
3 restraints
H-atom parameters constrained
Δρ_max_ = 0.79 e Å^−3^
Δρ_min_ = −0.41 e Å^−3^
Absolute structure: Assigned from the known absolute structure of the starting material; the Flack (1983 ▶) parameter is consistent with this assignment, 1119 Friedel pairs
Flack parameter: 0.12 (19)

### 

Data collection: *APEX2* (Bruker, 2007 ▶); cell refinement: *SAINT* (Bruker, 2007 ▶); data reduction: *SAINT*; program(s) used to solve structure: *SIR2004* (Burla *et al.*, 2005 ▶); program(s) used to refine structure: *SHELXL97* (Sheldrick, 2008 ▶); molecular graphics: *SHELXTL* (Sheldrick, 2008 ▶); software used to prepare material for publication: *SHELXTL*.

## Supplementary Material

Crystal structure: contains datablocks global, I. DOI: 10.1107/S1600536811016564/fy2002sup1.cif

Structure factors: contains datablocks I. DOI: 10.1107/S1600536811016564/fy2002Isup2.hkl

Supplementary material file. DOI: 10.1107/S1600536811016564/fy2002Isup3.cml

Additional supplementary materials:  crystallographic information; 3D view; checkCIF report

## Figures and Tables

**Table 1:** Hydrogen-bond geometry (Å, °)

*D*—H⋯*A*	*D*—H	H⋯*A*	*D*⋯*A*	*D*—H⋯*A*
O1—H1⋯O2^i^	0.82	1.83	2.641 (3)	168
N1—H1*A*⋯O3^ii^	0.9	1.83	2.713 (3)	166
N1—H1*B*⋯O1^iii^	0.9	2.14	2.916 (4)	144
N1—H1*B*⋯O2	0.9	2.35	2.687 (3)	102

Symmetry codes: (i) 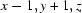; (ii) 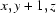; (iii) 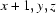.

**Fig. 1.**
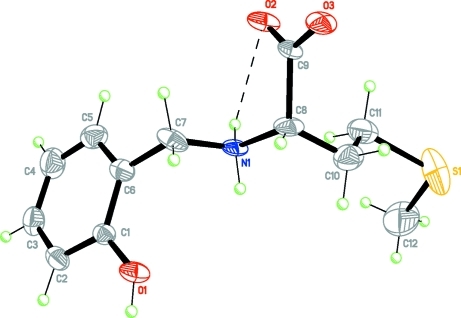
: ORTEP drawing with numbering of the atoms. Non H-atoms are represented as displacement ellipsoids plotted at the 50% probability level, while H-atoms bound to heteroatoms are shown as small spheres of arbitrary radius. The intramolecular H-bond is represented by a dashed line.

**Fig. 2.**
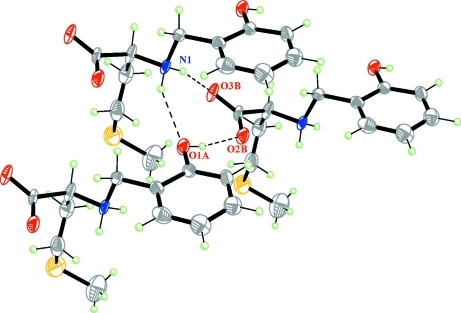
: Supramolecular arrangement of (I): a perpendicular view of the triangular-shaped ring [R23(8)], involving three molecules interconnected through the H-bonding interactions N1—H1A···O3, N1—H1B···O1 and O1—H1···O2. Dotted lines indicate H-bonding interactions.

**Fig. 3.**
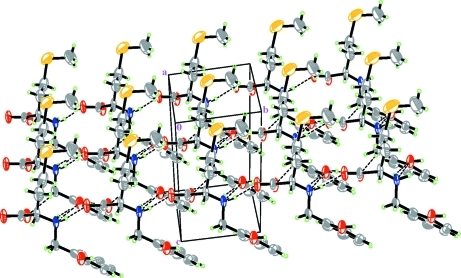
: Crystal packing of (I): molecular arrangement in parallel strings extending along the (001) crystallographic plane.

## supplementary crystallographic information

### Comment

Considerable attention has been devoted to both Schiff bases and reduced Schiff bases derived from salicylaldehyde and α-amino acids, since their transition metal complexes are closely analogous to the metal-free systems formed as intermediates in many reactions involving the vitamin B6, such as transamination, decarboxylation, α- and β-elimination and racemization (Martell, 1989). In this regard, copper(II) complexes of *N*-(2-hydroxybenzyl)-α-amino acids have been studied as models for the intermediate species that occur in the biological reactions mentioned above (Koh *et al.*, 1996) and such reduced Schiff bases have been used as chelating agents for organoboron (Nefkens *et al.*, 1985; Beltrán *et al.*, 2002) and transition metals such as copper (Koh *et al.*, 1996), zinc (Ritsma, 1975), cobalt (Bandyopadhyay *et al.*, 2006), nickel (Sreenivasulu *et al.*, 2004), manganese (Shongwe *et al.*, 1999), technetium (Wilson, 1990) and vanadium (Maurya, 2003). These ligands are more stable than the Schiff bases from which they originate and are suitable to provide conformationally flexible rings in complexation. This results in different solid state architectures, and the presence of hydrogen bond donors and acceptors enables the design and construction of supramolecular, three-dimensional networks (Ganguly *et al.*, 2008). This paper describes the structural characterization of the reduced Schiff base (*S*)-*N*-(2-hydroxybenzyl)methionine (I).

Figure 1 shows the molecular structure of (I). The crystal structure analysis clearly indicates that the amino acid is a pure enantiomer, since the compound crystallizes in the chiral *P*1 space group. In Figure 1 the L-enantiomer is shown with *S* absolute configuration at C8, in agreement with the synthetic precursor, L-(*S*)-methionine. The carboxylic acid has been found in the deprotonated —(COO)^-^ form, with the C9—O2 and C9—O3 bond distances being very similar. Meanwhile, N1 is protonated in the NH_2_^+^ form. Therefore compound (I) crystallizes as a zwitterion. The angle N1—C8—C9 is lower in comparison to the angles N1—C8—C10 and C10—C8—C9. This asymmetry in the angles at the C8 is due to the presence of an intramolecular interaction between the N—H and carboxylate groups forming a roughly planar five-membered ring containing H, N, Cα, C and O atoms in a pentagonal (C_5_) conformation.

A very interesting feature encountered in the crystal lattice of (I) is related to the formation of supramolecular triangle-shaped motifs [*R*^2^_3_(8)] involving molecules held together by O—H···O and N—H···O hydrogen bonds (Figure 2, Table 1). Other hydrogen bonding interactions govern the molecular arrangement in parallel strings extending along the (001) crystallographic plane (Figure 3).

### Experimental

The synthesis of the title compound (I) was performed according to the method previously employed for similar Schiff bases (Koh, *et al.*, 1996), starting from L-(S)-metionine. Recrystallization of (I) from a methanol solution produced single crystals suitable for X-ray diffraction.

### Refinement

H atoms were located in a difference Fourier map and placed in idealized positions using the riding-model technique, with distances C—H = 0.93–0.97 Å, N—H = 0.90 Å and with *U*_iso_(H) = 1.2*U*_eq_(C) or *U*_iso_(H) = 1.2*U*_eq_(N). The best refinement was obtained using the multi-scan *SADABS* (Sheldrick, 1996) correction. The restraints were generated automatically by *SHELXL97* to fix the origin.

### Crystal data

**Table:**

C_12_H_17_NO_3_S	*Z* = 1
*M**_r_* = 255.33	*F*(000) = 136
Triclinic, *P*1	*D*_x_ = 1.316 Mg m^−^^3^
Hall symbol: P 1	Mo *K*α radiation, λ = 0.71073 Å
*a* = 5.3221 (2) Å	Cell parameters from 4292 reflections
*b* = 5.8369 (2) Å	θ = 3.6–27.0°
*c* = 10.5564 (5) Å	µ = 0.25 mm^−^^1^
α = 98.200 (3)°	*T* = 296 K
β = 90.780 (3)°	Prysmatic, yellow
γ = 96.849 (3)°	0.53 × 0.44 × 0.16 mm
*V* = 322.10 (2) Å^3^	

### Data collection

**Table:**

Bruker APEXII CCD diffractometer	1966 reflections with *I* > 2σ(*I*)
graphite	*R*_int_ = 0.016
φ and ω scans	θ_max_ = 26°, θ_min_ = 3.8°
Absorption correction: multi-scan (*SADABS*; Sheldrick, 1996)	*h* = −6→6
*T*_min_ = 0.679, *T*_max_ = 0.746	*k* = −7→7
7430 measured reflections	*l* = −13→13
2324 independent reflections	

### Refinement

**Table:**

Refinement on *F*^2^	Secondary atom site location: difference Fourier map
Least-squares matrix: full	Hydrogen site location: inferred from neighbouring sites
*R*[*F*^2^ > 2σ(*F*^2^)] = 0.059	H-atom parameters constrained
*wR*(*F*^2^) = 0.179	*w* = 1/[σ^2^(*F*_o_^2^) + (0.1065*P*)^2^ + 0.1553*P*] where *P* = (*F*_o_^2^ + 2*F*_c_^2^)/3
*S* = 1.07	(Δ/σ)_max_ < 0.001
2324 reflections	Δρ_max_ = 0.79 e Å^−^^3^
156 parameters	Δρ_min_ = −0.41 e Å^−^^3^
3 restraints	Absolute structure: Assigned from the known absolute structure of the starting material; the Flack (1983) parameter is consistent with this assignment, 1119 Friedel-pairs
Primary atom site location: structure-invariant direct methods	Flack parameter: 0.12 (19)

### Special details

**Table:**

Geometry. All s.u.'s (except the s.u. in the dihedral angle between two l.s. planes) are estimated using the full covariance matrix. The cell s.u.'s are taken into account individually in the estimation of s.u.'s in distances, angles and torsion angles; correlations between s.u.'s in cell parameters are only used when they are defined by crystal symmetry. An approximate (isotropic) treatment of cell s.u.'s is used for estimating s.u.'s involving l.s. planes.
Refinement. Refinement of *F*^2^ against ALL reflections. The weighted *R*-factor *wR* and goodness of fit *S* are based on *F*^2^, conventional *R*-factors *R* are based on *F*, with *F* set to zero for negative *F*^2^. The threshold expression of *F*^2^ > 2σ(*F*^2^) is used only for calculating *R*-factors(gt) *etc*. and is not relevant to the choice of reflections for refinement. *R*-factors based on *F*^2^ are statistically about twice as large as those based on *F*, and *R*- factors based on ALL data will be even larger.

### Fractional atomic coordinates and isotropic or equivalent isotropic displacement parameters (Å^2^)

**Table:**

	*x*	*y*	*z*	*U*_iso_*/*U*_eq_	
C1	0.1397 (7)	0.8261 (5)	0.6478 (4)	0.0378 (8)	
C2	0.1286 (8)	1.0229 (6)	0.7378 (4)	0.0480 (10)	
H2	0.0037	1.1192	0.7301	0.058*	
C3	0.3045 (9)	1.0752 (9)	0.8391 (4)	0.0595 (11)	
H3	0.2993	1.2085	0.8984	0.071*	
C4	0.4846 (11)	0.9326 (10)	0.8521 (5)	0.0719 (14)	
H4	0.6064	0.9719	0.918	0.086*	
C5	0.4866 (8)	0.7305 (8)	0.7677 (5)	0.0592 (11)	
H5	0.6034	0.6289	0.7807	0.071*	
C6	0.3176 (7)	0.6750 (6)	0.6635 (4)	0.0437 (9)	
C7	0.3226 (7)	0.4586 (6)	0.5712 (5)	0.0523 (11)	
H7A	0.1526	0.4057	0.5364	0.063*	
H7B	0.3754	0.3381	0.6164	0.063*	
N1	0.4971 (5)	0.4903 (4)	0.4628 (3)	0.0365 (7)	
H1A	0.4664	0.6175	0.4288	0.044*	
H1B	0.6586	0.5129	0.4927	0.044*	
C12	0.982 (2)	0.7692 (13)	0.1065 (9)	0.125 (3)	
H12A	0.8321	0.8384	0.0902	0.187*	
H12B	1.1162	0.8272	0.0553	0.187*	
H12C	1.0313	0.8083	0.1955	0.187*	
C8	0.4605 (9)	0.2821 (6)	0.3620 (4)	0.0528 (11)	
H8	0.2775	0.234	0.3533	0.063*	
C9	0.5770 (7)	0.0784 (5)	0.4082 (4)	0.0396 (8)	
O1	−0.0230 (5)	0.7724 (4)	0.5440 (3)	0.0481 (7)	
H1	−0.0734	0.8918	0.5264	0.072*	
O2	0.7473 (5)	0.1274 (4)	0.4933 (3)	0.0542 (8)	
O3	0.4885 (6)	−0.1164 (4)	0.3543 (3)	0.0543 (8)	
S1	0.9203 (4)	0.4659 (3)	0.0671 (2)	0.1140 (8)	
C10	0.5426 (11)	0.3361 (8)	0.2347 (5)	0.0673 (14)	
H10A	0.4849	0.2056	0.1691	0.081*	
H10B	0.4698	0.472	0.2149	0.081*	
C11	0.8008 (11)	0.3782 (9)	0.2368 (6)	0.0749 (15)	
H11A	0.8727	0.24	0.2535	0.09*	
H11B	0.8581	0.5041	0.3051	0.09*	

### Atomic displacement parameters (Å^2^)

**Table:**

	*U*^11^	*U*^22^	*U*^33^	*U*^12^	*U*^13^	*U*^23^
C1	0.0352 (18)	0.0332 (17)	0.047 (2)	0.0050 (13)	0.0058 (17)	0.0120 (14)
C2	0.049 (2)	0.0382 (19)	0.060 (2)	0.0137 (15)	0.015 (2)	0.0095 (17)
C3	0.064 (3)	0.068 (3)	0.045 (2)	0.008 (2)	0.007 (2)	0.0004 (19)
C4	0.070 (3)	0.091 (4)	0.054 (3)	0.013 (3)	0.000 (3)	0.005 (3)
C5	0.047 (2)	0.071 (3)	0.065 (3)	0.013 (2)	0.002 (2)	0.025 (2)
C6	0.0355 (19)	0.0368 (18)	0.063 (2)	0.0053 (14)	0.0139 (19)	0.0199 (16)
C7	0.037 (2)	0.0255 (16)	0.096 (3)	0.0053 (13)	0.014 (2)	0.0138 (19)
N1	0.0462 (16)	0.0128 (11)	0.0509 (17)	0.0053 (10)	−0.0084 (14)	0.0058 (10)
C12	0.183 (9)	0.078 (4)	0.121 (6)	0.010 (5)	0.017 (6)	0.043 (4)
C8	0.079 (3)	0.0190 (15)	0.059 (2)	0.0112 (16)	−0.025 (2)	0.0016 (15)
C9	0.052 (2)	0.0163 (15)	0.0514 (19)	0.0096 (13)	0.0019 (19)	0.0044 (13)
O1	0.0499 (16)	0.0286 (12)	0.0668 (18)	0.0114 (10)	−0.0099 (15)	0.0056 (11)
O2	0.0539 (16)	0.0272 (12)	0.082 (2)	0.0108 (10)	−0.0169 (16)	0.0075 (12)
O3	0.081 (2)	0.0182 (12)	0.0622 (17)	0.0056 (11)	−0.0112 (16)	0.0037 (11)
S1	0.1068 (14)	0.1004 (13)	0.1180 (14)	−0.0026 (10)	0.0483 (12)	−0.0327 (11)
C10	0.092 (4)	0.046 (2)	0.064 (3)	0.011 (2)	−0.016 (3)	0.007 (2)
C11	0.087 (4)	0.057 (3)	0.078 (3)	0.026 (2)	−0.033 (3)	−0.009 (2)

### Geometric parameters (Å, °)

**Table:**

C1—O1	1.365 (5)	N1—H1B	0.9
C1—C2	1.389 (5)	C12—S1	1.749 (8)
C1—C6	1.392 (5)	C12—H12A	0.96
C2—C3	1.387 (7)	C12—H12B	0.96
C2—H2	0.93	C12—H12C	0.96
C3—C4	1.360 (7)	C8—C10	1.483 (7)
C3—H3	0.93	C8—C9	1.539 (4)
C4—C5	1.375 (7)	C8—H8	0.98
C4—H4	0.93	C9—O3	1.232 (4)
C5—C6	1.388 (6)	C9—O2	1.246 (5)
C5—H5	0.93	O1—H1	0.82
C6—C7	1.484 (6)	S1—C11	2.023 (7)
C7—N1	1.502 (5)	C10—C11	1.366 (8)
C7—H7A	0.97	C10—H10A	0.97
C7—H7B	0.97	C10—H10B	0.97
N1—C8	1.489 (4)	C11—H11A	0.97
N1—H1A	0.9	C11—H11B	0.97
			
O1—C1—C2	121.9 (3)	S1—C12—H12A	109.5
O1—C1—C6	118.1 (3)	S1—C12—H12B	109.5
C2—C1—C6	120.0 (3)	H12A—C12—H12B	109.5
C3—C2—C1	119.7 (4)	S1—C12—H12C	109.5
C3—C2—H2	120.1	H12A—C12—H12C	109.5
C1—C2—H2	120.1	H12B—C12—H12C	109.5
C4—C3—C2	120.3 (4)	C10—C8—N1	112.6 (3)
C4—C3—H3	119.8	C10—C8—C9	115.0 (4)
C2—C3—H3	119.8	N1—C8—C9	109.9 (3)
C3—C4—C5	120.0 (5)	C10—C8—H8	106.2
C3—C4—H4	120	N1—C8—H8	106.2
C5—C4—H4	120	C9—C8—H8	106.2
C4—C5—C6	121.2 (4)	O3—C9—O2	127.9 (3)
C4—C5—H5	119.4	O3—C9—C8	114.6 (3)
C6—C5—H5	119.4	O2—C9—C8	117.5 (3)
C5—C6—C1	118.4 (4)	C1—O1—H1	109.5
C5—C6—C7	121.2 (4)	C12—S1—C11	100.5 (3)
C1—C6—C7	120.4 (4)	C11—C10—C8	108.8 (4)
C6—C7—N1	113.2 (3)	C11—C10—H10A	109.9
C6—C7—H7A	108.9	C8—C10—H10A	109.9
N1—C7—H7A	108.9	C11—C10—H10B	109.9
C6—C7—H7B	108.9	C8—C10—H10B	109.9
N1—C7—H7B	108.9	H10A—C10—H10B	108.3
H7A—C7—H7B	107.8	C10—C11—S1	110.1 (4)
C8—N1—C7	110.7 (3)	C10—C11—H11A	109.6
C8—N1—H1A	109.5	S1—C11—H11A	109.6
C7—N1—H1A	109.5	C10—C11—H11B	109.6
C8—N1—H1B	109.5	S1—C11—H11B	109.6
C7—N1—H1B	109.5	H11A—C11—H11B	108.1
H1A—N1—H1B	108.1		
			
O1—C1—C2—C3	−177.5 (4)	C1—C6—C7—N1	91.8 (4)
C6—C1—C2—C3	3.7 (6)	C6—C7—N1—C8	−169.9 (3)
C1—C2—C3—C4	−1.2 (7)	C7—N1—C8—C10	156.6 (4)
C2—C3—C4—C5	−2.7 (8)	C7—N1—C8—C9	−73.8 (4)
C3—C4—C5—C6	4.3 (8)	C10—C8—C9—O3	−74.6 (5)
C4—C5—C6—C1	−1.8 (6)	N1—C8—C9—O3	157.1 (3)
C4—C5—C6—C7	179.0 (5)	C10—C8—C9—O2	105.0 (4)
O1—C1—C6—C5	178.9 (4)	N1—C8—C9—O2	−23.4 (5)
C2—C1—C6—C5	−2.2 (5)	N1—C8—C10—C11	71.0 (5)
O1—C1—C6—C7	−1.8 (5)	C9—C8—C10—C11	−55.9 (5)
C2—C1—C6—C7	177.1 (4)	C8—C10—C11—S1	−177.7 (3)
C5—C6—C7—N1	−89.0 (5)	C12—S1—C11—C10	100.9 (5)

### Hydrogen-bond geometry (Å, °)

**Table:**

*D*—H···*A*	*D*—H	H···*A*	*D*···*A*	*D*—H···*A*
O1—H1···O2^i^	0.82	1.83	2.641 (3)	168
N1—H1A···O3^ii^	0.9	1.83	2.713 (3)	166
N1—H1B···O1^iii^	0.9	2.14	2.916 (4)	144
N1—H1B···O2	0.9	2.35	2.687 (3)	102

Symmetry codes: (i) *x*−1, *y*+1, *z*; (ii) *x*, *y*+1, *z*; (iii) *x*+1, *y*, *z*.
